# Non‐SMC condensin I complex subunit H enhances proliferation, migration, and invasion of hepatocellular carcinoma

**DOI:** 10.1002/mc.23114

**Published:** 2019-09-15

**Authors:** Chengjun Sun, Shanzhou Huang, Hanyu Wang, Rongxing Xie, Lishan Zhang, Qi Zhou, Xiaoshun He, Weiqiang Ju

**Affiliations:** ^1^ Organ Transplant Center, The First Affiliated Hospital Sun Yat‐sen University Guangzhou China; ^2^ Department of Liver Surgery, The First Affiliated Hospital Sun Yat‐Sen University Guangzhou China; ^3^ Department of General Surgery, Hui Ya Hospital of The First Affiliated Hospital Sun Yat‐sen University Huizhou Guangdong China; ^4^ Department of General Surgery, Guangdong General Hospital Guangdong Academy of Medical Sciences Guangdong China

**Keywords:** hepatocellular carcinoma, invasion, migration, NCAPH, prognosis

## Abstract

Non‐SMC condensing I complex subunit H (NCAPH) is a member of the Barr protein family and part of the condensin I complex. The upregulation of NCAPH is associated with poor prognosis in patients with colon cancer. However, the relationship between NCAPH and hepatocellular carcinoma (HCC) remains unclear. This study aimed to explore NCAPH expression in HCC tissues and to investigate NCAPH functions in HCC cells. In this study, we found that high expression of NCAPH in HCC indicated worse prognosis via bioinformatics analysis. Consistently, quantitative real‐time polymerase chain reaction assays in 20 pairs of HCC specimens and the immunohistochemical analysis of 100 HCC tissues showed the upregulation of NCAPH. We established stable NCAPH‐overexpressing and NCAPH knockdown cell lines. Cell Counting Kit‐8 assays and colony formation assay were performed to analyze cell proliferation. Migration and invasion were analyzed by Transwell assays. Subcutaneous xenograft models were used to explore the role of NCAPH in tumor formation in vivo. Our results showed that NCAPH promoted tumor proliferation, migration, and invasion in vitro and in vivo. In conclusion, our findings indicate that NCAPH could serve as a novel prognostic biomarker and a potential therapeutic target for patients with HCC.

**Abbreviations**
ANLTs: adjacent normal liver tissues
CCK‐8: cell counting kit‐8
CFA: colony formation assay
DFS: disease‐free survival
GEO: Gene Expression Omnibus
HCC: hepatocellular carcinoma
IHC: immunohistochemistry
NCAPH: non‐SMC condensin I complex subunit H
OS: overall survival
OV: overexpression
qRT‐PCR: quantitative real‐time polymerase chain reaction
TCGA: The Cancer Genome Atlas

## 1 INTRODUCTION

Hepatocellular carcinoma (HCC) is still the third leading cause of death related to cancer due to the high mortality caused by poor prognosis and frequent metastasis and relapse.^1^, ^2^, ^3^ Although HCC patients can be treated by surgery, radiofrequency ablation, and liver transplantation, the 5‐year survival rate of HCC patients remains low.^1^ Therefore, the identification of genes that may contribute to novel treatments and prolong the survival of HCC patients is urgently needed.

Condensin is important for chromosome assembly and segregation during mitosis and meiosis.^4^ Many eukaryotic cells contain condensin I and condensin II, which are composed of sets of subunits.^5^ The condensin I complex comprises structural maintenance of chromosome (SMC) proteins and three non‐SMC subunits, including non‐SMC condensing I complex subunit H (NCAPH), non‐SMC condensing I complex subunit G, and non‐SMC condensing I complex subunit D2.^6^ Previous studies have reported that the abnormal expression of the non‐SMC condensin I complex is involved in the progression of human cancer.^7^, ^8^ NCAPH encodes a regulatory subunit in the non‐SMC condensing I complex and is required for the conversion of interphase chromatin into condensed chromosomes.^9^ Previous studies have revealed that abnormal expression of NCAPH is associated with colon cancer and prostate cancer.^10^, ^11^ However, there have been no relevant reports about the clinical relevance and functional role of NCAPH in HCC. In this study, we aimed to explore NCAPH expression in HCC tissues and investigate the functions of NCAPH in HCC cells.

## 2 MATERIALS AND METHODS

### 2.1 Patients and clinicopathological data

All tissue samples and adjacent nontumor tissue specimens were collected from 100 HCC patients who underwent hepatectomy at the First Affiliated Hospital of Sun Yat‐sen University (Guangzhou, China) between July 2013 and December 2014. None of the patients received radiotherapy or preoperative chemotherapy before surgery. All tissues were histopathologically confirmed, and all patients were followed up until December 2019. All fresh samples were stored at −80℃ immediately after resection. Detailed information on the clinical characteristics of all patients is documented in Table 1. Our study was approved by the Ethics Committee of the First Affiliated Hospital of Sun Yat‐sen University and conformed to the 1964 Declaration of Helsinki and its later amendments or comparable ethical standards. Clinical samples were collected from patients after written informed consent was obtained.

**Table 1:** Correlation between NCAPH expression with clinicopathological characteristics of HCC

Clinicopathological Variables	n	NCAPH Expression		*P*
		Low (39)	High (61)	
Sex				
Male	86	34	52	
Female	14	5	9	.79
Age, years				
<50	51	20	31	
≥50	49	19	30	.96
HBsAg				
Negative	40	17	23	.56
Positive	60	22	38	
AFP, ng/L				
<200	48	24	24	**.03**
≥200	52	15	37	
Tumor size, cm				
≤5	50	27	23	**<.01**
>5	50	12	38	
Tumor number				
Solitary	57	28	29	**.02**
Multiple (≥2)	43	11	32	
PVTT				
Absence	65	34	31	**<.01**
Presence	35	5	30	
TNM stage				
Early (I & II)	52	32	20	
Late (III & IV)	48	7	41	**<.01**
Differentiation grade				
Well	64	36	28	
Poor	36	3	33	**<.01**

Abbreviations: AFP, alpha fetoprotein; HBsAg, hepatitis B surface antigen; NCAPH, non‐SMC condensin I complex subunit H; PVTT, portal vein tumor thrombus.

### 2.2 High‐throughput data processing

Detailed data on NCAPH expression in HCC were downloaded from The Cancer Genome Atlas (TCGA, http://gdc.cancer.gov/). The data used in the microarray were downloaded from the Gene Expression Omnibus (GEO) datasets GSE6764, GSE29721, GSE45436, GSE62232, and GSE84402 (http://www.ncbi.nlm.nih.gov/geo). The data from TCGA and GEO were log_2_‐transformed, and the results were analyzed using Excel 2018 and the GraphPad Prism 6 software.

Gene set enrichment analysis (GSEA) was performed to investigative pathways associated with NCAPH using the data obtained from TCGA. GSEA is supported by the Broad Institute Website (http://software.broadinstitute.org/gsea/index.jsp).

### 2.3 Hematoxylin and eosin staining

Tissue samples consisting of 100 pairs of HCC tumors together with matched adjacent normal tissues were fixed in formalin and embedded in paraffin for NCAPH immunohistochemistry (IHC). The scores were evaluated via two scoring systems: the positive cell score and staining intensity score. The NCAPH antibodies used for Western blotting and IHC were obtained from Proteintech (11515‐1‐AP; Chicago, IL).

### 2.4 RNA extraction and quantitative real‐time polymerase chain reaction

Total RNA was extracted with TRIzol reagent (Invitrogen, NY) according to the manufacturer's protocol. Quantitative real‐time polymerase chain reaction (qRT‐PCR) was performed using the SYBR green detection RT‐PCR system (Takara, Japan) with primers for NCAPH (forward primer: AAACAACCTCAATGTCTCCGAAG; reverse primer: ACAACCTAACTCTGGCAACTCG) and β‐actin (forward primer: CACCCAGCACAATGAAGATCAAGAT; reverse primer: CCAGTTTTTAAATCCTGAGTCAAGC) (Servicebio Technology, Wuhan, China). The β‐actin gene was used as the reference control. The relative messenger RNA (mRNA) expression was quantified using the $2^{-\Delta \Delta C_{t}}$ method.

### 2.5 Cell culture

Human HCC cell lines, including LM3, Huh7, PLC/PRF/5, and Hep3B were purchased from the Institute of Biochemistry and Cell Biology (Chinese Academy of Sciences, Shanghai, China). HCC cell lines were incubated in DMEM (Gibco, Gaithersburg) with 10% fetal bovine serum and maintained at 37℃ in a humidified incubator with controlled temperature and 5% CO_2_.

### 2.6 Lentivirus production and stable cell line construction

Lentiviral vectors expressing short hairpin RNA (shRNA) and NCAPH were cotransfected with the packaging vectors psPAX2 and pMD2G (Addgene) into HEK293FT cells for lentivirus production using Lipofectamine 3000 in accordance with the manufacturer's instructions. To establish stable cell lines, cells were transduced by using the above lentiviruses with polybrene (8 mg/mL, Sigma). After incubating for 72 hours, cells were selected with 2 mg/mL puromycin for 3 days.

### 2.7 Western blotting

Cells were lysed in cold radioimmunoprecipitation assay buffer containing protease inhibitors. Protein concentrations were measured by a BCA Protein Quantitation Assay (KeyGen Biotech, Nanjing, China). Total protein was transferred to a nitrocellulose membrane after denaturation in a 10% sodium dodecyl sulfate polyacrylamide gel electrophoresis gel. After incubation with primary antibodies and secondary antibodies, the targeted proteins were detected using ECL reagents (EMD Millipore, MA). Primary antibodies against E‐cadherin (1:1000; Abcam, Cambridge, MA), vimentin (1:1000; Abcam) and N‐cadherin (1:1000; Abcam) were used.

### 2.8 Cell proliferation assay

A Cell Counting Kit‐8 assay (CCK‐8; Dojindo, Tabaru, Japan) was used to measure cell proliferation. Five hundred cells were seeded into each well of a 96‐well plate. The absorption values were measured at the following time points: 24, 48, and 72 hours after shRNA transfection.

### 2.9 Colony formation assay

Transected HCC cells were seeded in a six‐well plate at a density of 600 cells. Cell colonies were fixed with 4% paraformaldehyde and stained with 1% crystal violet. Colonies were examined and counted under microscope.

### 2.10 Transwell assay

Migration and invasion were assessed using Transwell plates. For the migration assay, 5 × 10^4^ cells were plated in each chamber. For the invasion assay, 5 × 10^4^ cells were resuspended in 250 µL of unsupplemented medium in the upper chamber (Corning, NY), while the lower chamber was filled with 0.75 mL of complete medium. The upper chamber was fixed with 4% methanol and stained with 0.1% crystal violet stain. The transmembrane cells were estimated under a microscope (Nikon, Tokyo, Japan) at ×200 magnification.

### 2.11 Animal experiments

Animal experiments were performed according to the protocol filed with the Guidance of Institutional Animal Care and Use Committee of Sun Yat‐sen University and with the approval of the Institutional Review Board of Sun Yat‐sen University. A total of 2 × 10^6^ Huh7 cells were subcutaneously injected into the nude mice. The tumor volume was measured every 7 days. Twenty‐eight days after implantation, the mice were euthanized by cervical dislocation and the tumor were excised, fixed weighted, and stored.

### 2.12 Statistical analysis

All data analyses were performed with the SPSS 22.0 statistical software. Cox regression and Kaplan‐Meier methods were used to analyze overall and disease‐free survival (DFS). The significance of the in vitro data results was determined by applying the Student *t* test. All experiments were repeated at least three times. *P* < .05 was considered to indicate a significant difference from the control.

## 3 RESULTS

### 3.1 NCAPH is upregulated in human HCC tissues and serves as an independent prognostic marker in HCC patients

**Figure 1:**
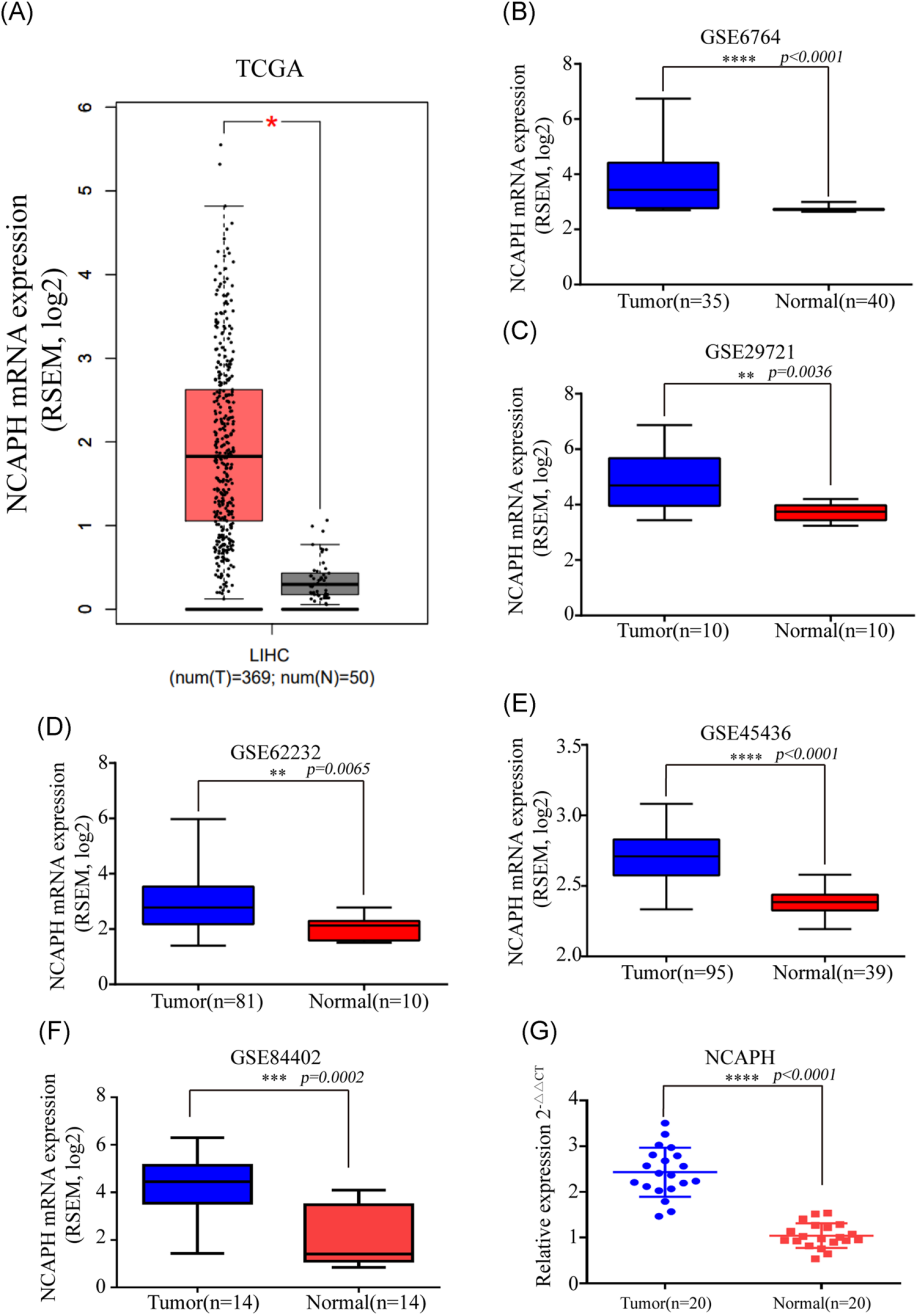
NCAPH is upregulated in HCC tissues. NCAPH was highly expressed in HCC tissues compared with normal liver tissues in the TCGA dataset (A; tumor, n = 369; normal, n = 50, *P < *.05) and GEO (B‐F; GSE6764, tumor, n = 35; normal, n = 40, *P < *.0001; GSE29721, tumor, n = 10; normal, n = 10, *P = *.0036; GSE45436, tumor, n = 95; normal, n = 39, *P < *.0001; GSE62232, tumor, n = 81; normal, n = 10, *P = *.0065; GSE84402, tumor, n = 14; normal, n = 14, *P = *.0002). NCAPH expression was upregulated in HCC upon comparison of 20 pairs of HCC tissues and ANLTs (*P < *.0001) (G). *Statistical significance. The Student *t* test was used to compare the differences. HCC, hepatocellular carcinoma; NCAPH, non‐SMC condensin I complex subunit H [Color figure can be viewed at wileyonlinelibrary.com]

**Figure 2:**
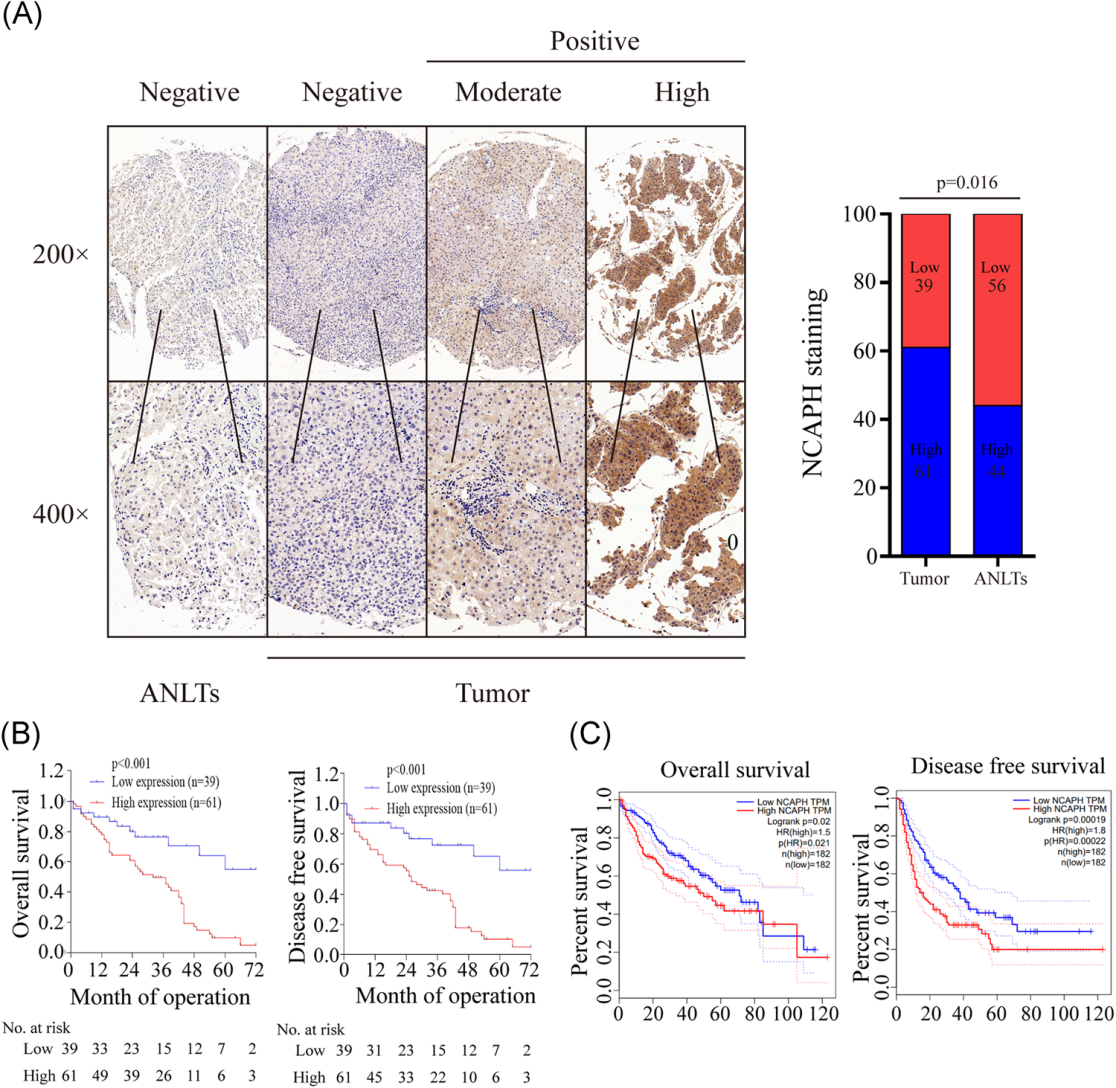
NCAPH expression is associated with poor prognosis. A, Immunohistochemical staining showed low NCAPH expression in normal liver tissues and high expression in HCC tissues. The scale bars indicate 50 and 20 μm. The NCAPH expression was high in HCC tissues compared with ANLTs (*P =* 0.016). B, Overall survival and disease‐free survival curves for the HCC patient groups showed increased NCAPH expression in patients with poor prognosis. C, Overall survival and disease‐free survival analysis according to GEPIA data indicated that patients with high NCAPH expression had decreased survival. *Statistical significance. The Student *t* test was used to compare the differences. ANLTs, adjacent normal liver tissues; GEPIA, Gene Expression Profiling Interactive Analysis; HCC, hepatocellular carcinoma; NCAPH, non‐SMC condensin I complex subunit H [Color figure can be viewed at wileyonlinelibrary.com]

To examine NCAPH expression in HCC, we analyzed NCAPH expression by using mRNA sequencing or microarray datasets from TCGA and GEO (GSE6764, GSE29721, GSE45436, GSE62232, and GSE84402). The results indicated that NCAPH expression was significantly increased in these datasets (Figure 1A‐F). The qRT‐PCR results showed that NCAPH expression was significantly higher in HCC tissues than in normal tissues (1G). In addition, the IHC results demonstrated that the NCAPH immunostaining signals were stronger in HCC tissues than in adjacent normal liver tissues (ANLTs; Figure 2A). Furthermore, we analyzed the association between NCAPH expression and the clinicopathological characteristics of HCC patients and found that NCAPH expression was positively related to alpha fetoprotein, tumor size, tumor number, portal vein thrombus, TNM stage, and differentiation grade (Table 1). Multivariate analysis demonstrated that NCAPH expression was a dependent prognostic factor for overall survival (OS; Table 2) and that NCAPH was an independent prognostic factor for DFS in HCC patients (Table 3). Then, we analyzed the association of NCAPH expression with survival and found that patients with a high NCAPH expression level had decreased OS and DFS compared to those with a low NCAPH level (Figure 2B). Consistently, the Gene Expression Profiling Interactive Analysis dataset results revealed that patients with a high NCAPH expression level exhibited lower OS and DFS than those with low NCAPH levels (Figure 2C).

**Table 2:** Univariate and multivariate Cox regression analysis of risk factors associated with overall survival

Variables	Univariate analysis			Multivariate analysis		
	HR	95% CI	*P*	HR	95% CI	*P*
NCAPH expression (high vs low)	3.51	1.82‐6.78	**<.01**	2.62	1.22‐5.63	**.01**
Sex (male vs female)	2.32	0.98‐5.46	.06			
Age (≥50 vs <50)	1.07	0.65‐1.77	.79			
HBsAg (positive vs negative)	1.28	0.75‐2.19	.36			
AFP (≥200 ng/mL vs <200 ng/mL)	1.60	0.95‐2.70	.08			
Tumor size (>5 cm vs ≤5 cm)	3.73	2.10‐6.62	**<.01**	2.97	1.52‐5.80	**<.01**
Tumor number (multiple vs single)	1.46	0.88‐2.41	.15			
PVTT (presence vs absence)	2.37	1.44‐3.93	**<.01**	1.03	0.53‐2.02	.93
TNM stage (late vs early)	3.02	1.78‐5.14	**<.01**	1.67	0.79‐3.51	.18
Differentiation grade (poor vs well)	2.20	1.33‐3.64	**<.01**	1.55	0.77‐3.09	.22

Abbreviation: AFP, alpha fetoprotein; HBsAg, hepatitis B surface antigen; NCAPH, non‐SMC condensin I complex subunit H; PVTT, portal vein tumor thrombus.

**Table 3:** Univariate and multivariate Cox regression analysis of risk factors associated with disease‐free survival

Variables	Univariate analysis			Multivariate analysis		
	HR	95% CI	*P*	HR	95% CI	*P*
NCAPH expression (high vs low)	3.47	1.79‐6.73	**<.01**	2.40	1.11‐5.19	**.03**
Sex (male vs female)	2.11	0.89‐5.00	.09			
Age (≥50 vs <50)	1.02	0.61‐1.71	.94			
HBsAg (positive vs negative)	1.34	0.77‐2.31	.30			
AFP (≥200 ng/mL vs <200 ng/mL)	1.64	0.95‐2.80	.07			
Tumor size (>5 cm vs ≤5 cm)	4.34	2.37‐7.94	**<.01**	3.64	1.82‐7.29	**<.01**
Tumor number (multiple vs single)	1.58	0.94‐2.65	.09			
PVTT (presence vs absence)	2.35	1.40‐3.93	**<.01**	0.84	0.42‐1.65	.61
TNM stage (late vs early)	3.41	1.96‐5.92	**<.01**	2.10	0.96‐4.56	.06
Differentiation grade (poor vs well)	2.31	1.38‐3.87	**<.01**	1.62	0.79‐3.30	.19

Abbreviation: AFP, alpha fetoprotein; HBsAg, hepatitis B surface antigen; NCAPH, non‐SMC condensin I complex subunit H; PVTT, portal vein tumor thrombus.

### 3.2 NCAPH promotes the proliferation of HCC cells in vitro

**Figure 3:**
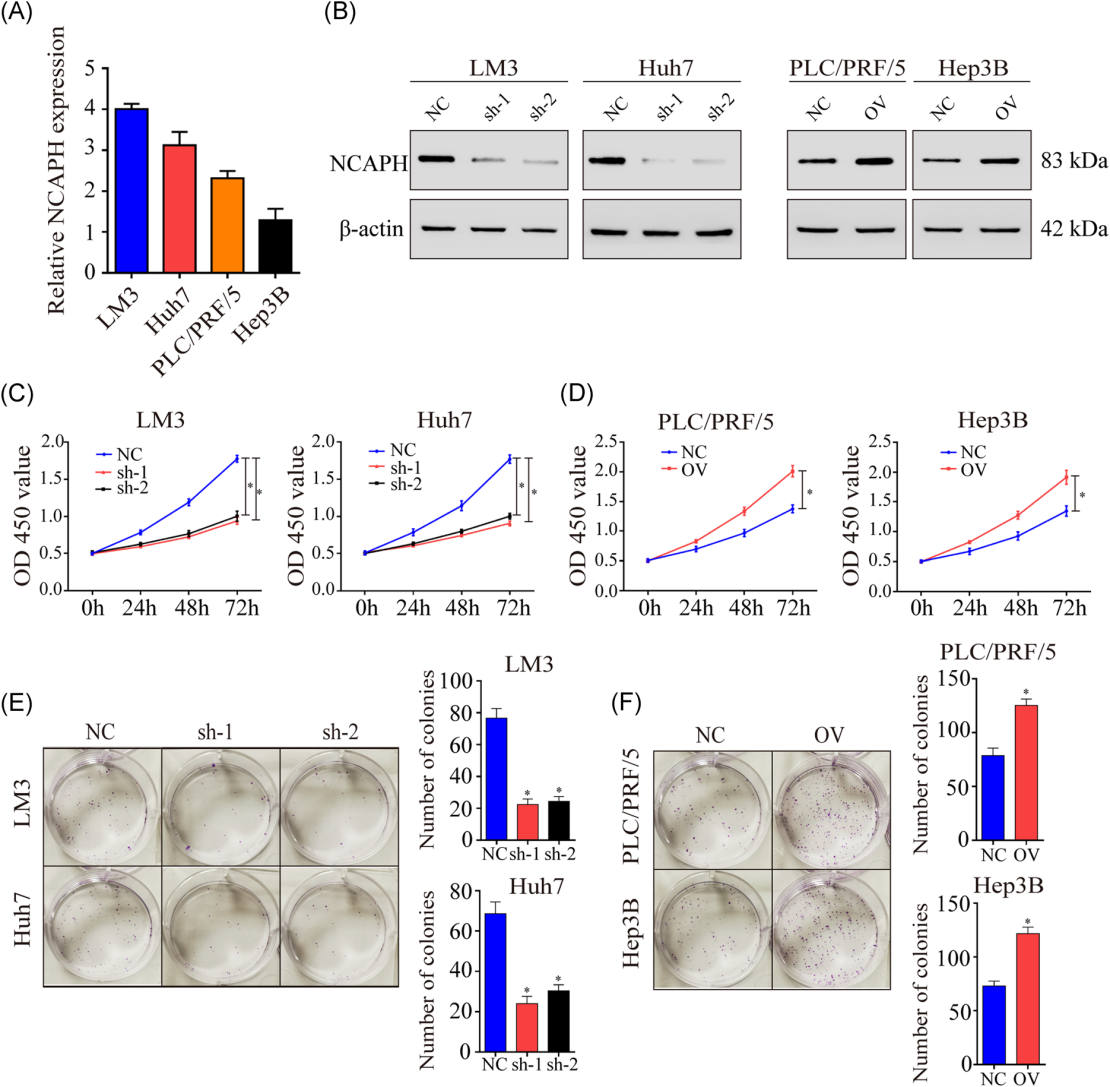
NCAPH affects HCC cell proliferation. A, NCAPH expression was detected in LM3, Huh7, PLC/PRF/5, and Hep3B cells. B, NCAPH expression were decreased in LM3 and Huh7 cells after transfected shRNAs, and NCAPH expression were increased in PLC/PRF/5 and Hep3B cells after overexpressed NCAPH. C, Knockdown of NCAPH suppressed LM3 and Huh7 cells proliferation by using CCK‐8 assays after 72 hours (*P < *.001). D, Overexpression of NCAPH promoted PLC/PRF/5 and Hep3B cells proliferation by using CCK‐8 assays after 72 hours (*P < *.001). E, The results of CFA assays showed that silencing NCAPH expression inhibited LM3 and Huh7 cells proliferation. F, The results of CFA assays showed that overexpressed NCAPH expression promoted PLC/PRF/5 and Hep3B cells proliferation. *Statistical significance. The Student *t* test was used to compare the differences. CCK‐8, cell counting kit‐8; CFA, colony formation assay; NCAPH, non‐SMC condensin I complex subunit H; shRNA, short hairpin RNA [Color figure can be viewed at wileyonlinelibrary.com]

To explore the biological function of NCAPH, we detected its mRNA levels in HCC cell lines (Figure 3A). Then, we established a stable knockdown LM3 and Huh7 cell lines and NCAPH‐overexpressing PLC/PRF/5 and Hep3B cell lines. Then, the protein levels of NCAPH in the different cell lines were tested by Western blot (Figure 3B). To determine whether NCAPH plays an important role in HCC cell proliferation, we used a CCK‐8 assay and colony formation assay (CFA) assay. CCK‐8 and CFA showed that NCAPH knockdown prominently suppressed the proliferation of HCC cell lines (Figure 3C and 3E), while NCAPH overexpression (OV) promoted the proliferation of HCC cells (Figure 3D and 3F). Overall, NCAPH promotes the proliferation of HCC cells.

### 3.3 NCAPH promotes the migration and invasion of HCC

**Figure 4:**
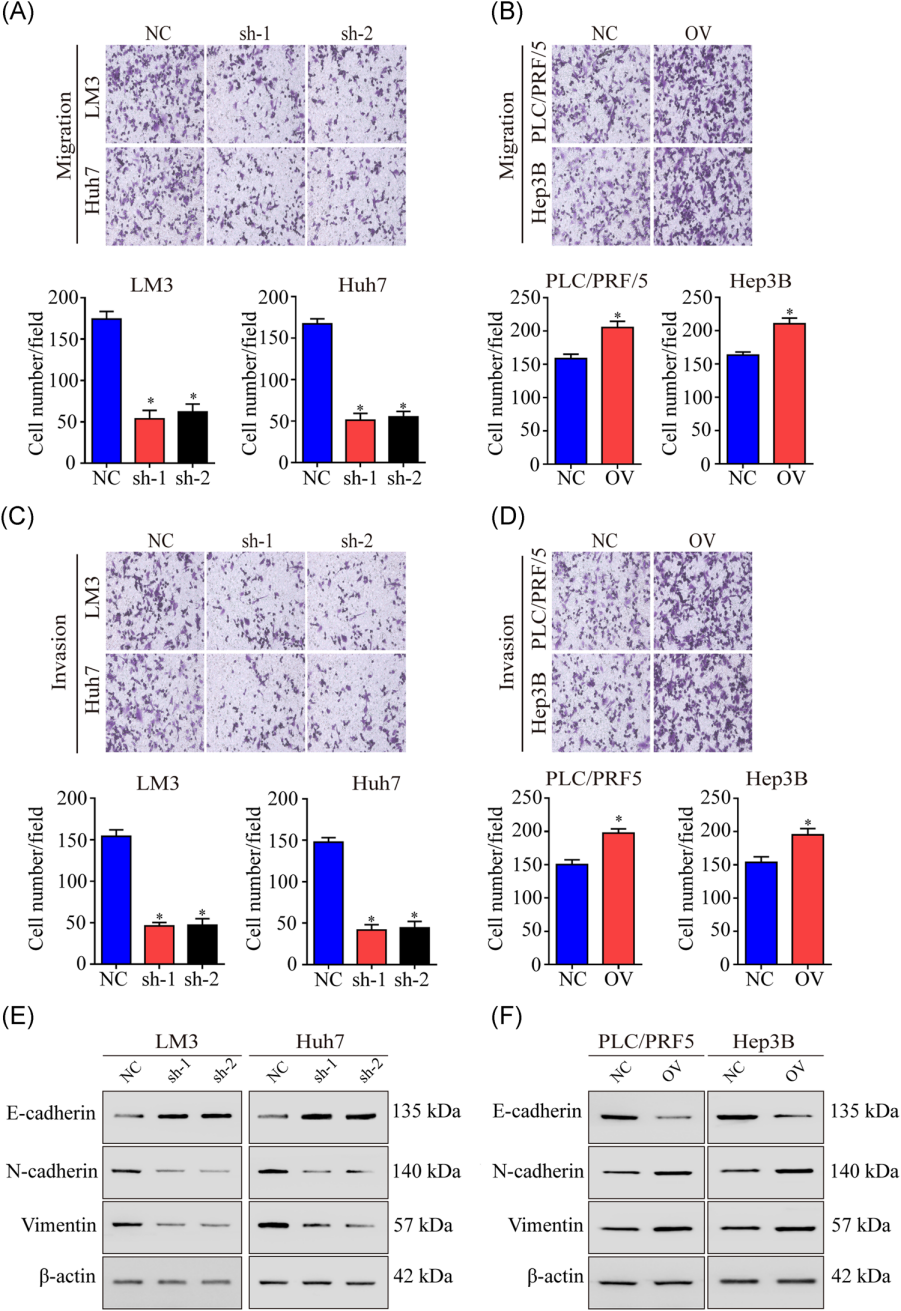
NCAPH affects motility in HCC cells. A, Knockdown of NCAPH reduces the migration in LM3 and Huh7 cells (*P < *.001). B, NCAPH overexpression promotes the migrated ability of LM3 and Huh7 cells (*P < *.001). C, Invasion chamber results showed that silencing NCAPH decreased the invaded ability of PLC/PRF/5 and Hep3B cells (*P < *.001). D, The invasion chamber results showed that overexpressing NCAPH expression could increase the PLC/PRF/5 and Hep3B cells invaded ability (*P < *.001). E, Silencing of NCAPH expression reduced the expression levels of N‐cadherin and Vimentin while increasing E‐cadherin expression in LM3 and Huh7 cells. F, Overexpression of NCAPH increased the levels of N‐cadherin and Vimentin but decreased E‐cadherin expression in PLC/PRF/5 and Hep3B cells. *Statistical significance. The Student *t* test was used to compare the differences. NCAPH, non‐SMC condensin I complex subunit H [Color figure can be viewed at wileyonlinelibrary.com]

Metastasis and recurrence are the most important causes of death in patients with HCC. We next examined the function of NCAPH in migration and invasion. The results showed that NCAPH knockdown had a significant inhibitory effect on migration and invasion compared to that resulting from shNC transfection, as demonstrated by the Transwell assay and invasion chamber assay (Figure 4A and 4B). NCAPH OV promoted cell migration and invasion in HCC cells. These results indicated that NCAPH promoted HCC migration and invasion (Figure 4C and 4D).

### 3.4 NCAPH promotes the expression of mesenchymal markers and proliferation markers in HCC

In previous studies, we found that NCAPH promotes proliferation, migration and invasion in HCC. Epithelial‐mesenchymal transition (EMT) markers are common markers reflecting cell mesenchymal or epithelial status. In our study, we performed the GSEA analysis and found that NCAPH was associate with the adherens junction pathway (Figure S1A). In addition, we found that the expression of NCAPH was associated with slug expression in TCGA (Figure S1B). Next, we detected the relationship between E‐cadherin, N‐cadherin, and vimentin with NCAPH. The results showed that the NCAPH expression was negative correlated with E‐cadherin expression, but positive correlated with N‐cadherin and vimentin expression (Figure S1C‐E). Moreover, we detected the expression of EMT markers in the NCAPH knockdown cell lines and the NCAPH‐overexpressing cell line. The results showed that N‐cadherin and vimentin were decreased, but E‐cadherin was increased in the shNCAPH cell lines (Figure 4E). However, mesenchymal markers such as N‐cadherin and vimentin were increased and epithelial markers such as E‐cadherin were decreased in the NCAPH‐overexpressing cell line (Figure 4F). Overall, NCAPH promotes the expression of mesenchymal markers and proliferation markers in HCC.

### 3.5 Knockdown of NCAPH suppresses tumor growth in vivo

**Figure 5:**
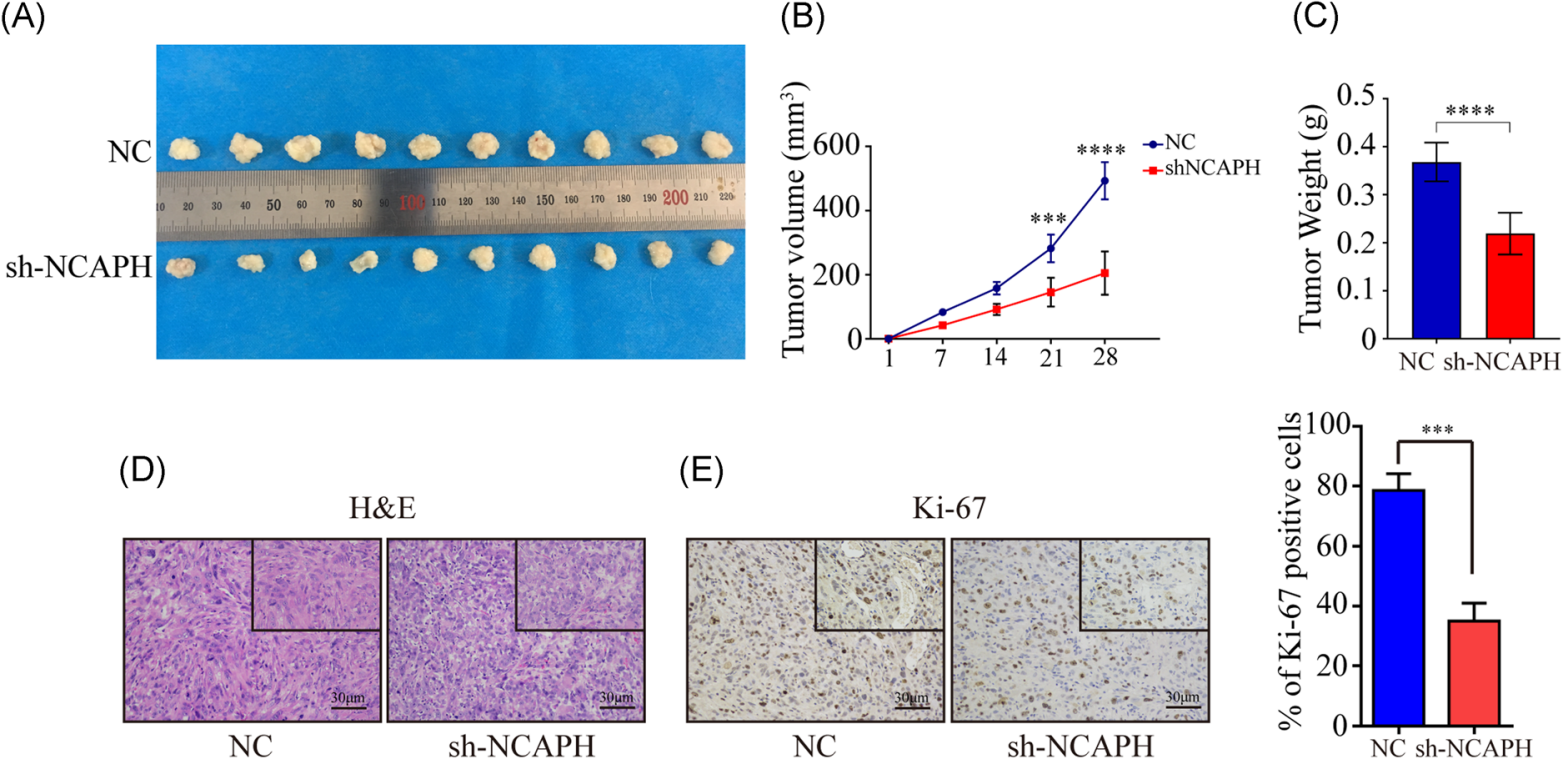
Silencing of NCAPH represses tumor growth in vivo. A, A total of 2 × 10^6^ Huh7 cells stably expressing NCAPH shRNAs and NC were implanted into nude mice. B, Tumor weight were decreased in the sh‐NCAPH group compared with that in the NC group (*P < *.001). C, The tumor volumes were decreased after transfected shRNAs compared with NC group (*P < *.001). D, H&E staining of tumors in the sh‐NCAPH group and NC group. E, The percentage of Ki‐67 staining were decreased in sh‐NCAPH group compared with NC group (*P < *.001). *Statistical significance. The Student *t* test was used to compare the differences. NCAPH, non‐SMC condensin I complex subunit H; NC, negative control; shRNA, short hairpin RNA [Color figure can be viewed at wileyonlinelibrary.com]

To further validate the tumor‐promoting function of NCAPH, we performed an in vivo xenograft assay using nude mice. NCAPH knockdown significantly reduced tumor growth compared with that observed in control cells (Figure 5A). Tumor weight and volume were significantly decreased in NCAPH knockdown cells (Figure 5B and 5C). In addition, IHC staining showed that the expression of the proliferation marker gene Ki‐67 was significantly decreased in NCAPH knockdown tumors (Figure 5D and 5E). These findings indicated that the knockdown of NCAPH inhibited tumorigenesis in vivo.

## 4 DISCUSSION

Increasing evidence confirms that abnormalities in the non‐SMC condensin I complex are associated with the progression of HCC.^8^ NCAPH is one of the three non‐SMC subunits in the condensin I complex and plays an essential role in condensin complex stability and the resolution of sister chromatids.^9^ To the best of our knowledge, our study presents the first evidence that NCAPH expression is upregulated in HCC tissues and cell lines, which is consistent with the data from the GEO dataset. In addition, the IHC assay indicated that the expression of NCAPH is significantly associated with clinicopathological features and reduced survival in patients with HCC. These findings suggest that NCAPH may be involved in HCC progression.

The condensin I complex plays an important role in chromosome condensation and segregation.^12^, ^13^ The aberrant expression of the subunits of the complex causes incomplete chromosome condensation.^14^ NCAPH is a part of the condensin complex. A recent study showed that NCAPH is upregulated in colon cancer and associated with poor prognosis. Previous studies have reveals that NCAPH transcript is present in proliferating cells and preceded by phosphorylation of histone H3 at Ser‐10.^15^, ^16^ Various studies suggested that phosphorylation of histone H3 serine contributed to the HCC progression.^17^ Thus, we hypothesis that NCAPH could promotes HCC progression. First, we detected the expression of NCAPH in HCC tissue and ANLTs, and found NCAPH was increased in HCC tissues compared with ANLTs. Then we analyzed the relationship between NCAPH expression and prognosis. The results demonstrated that the aberrant expression of NCAPH is associated with poor survival in patients with HCC. However, the role of NCAPH in HCC cells remains unexplored. In this study, we found that the proliferation ability of HCC cells was significantly reduced in NCAPH knockdown cells compared with that in the control group cells by CCK‐8 and CFA assay. To further verify our results, we established NCAPH‐overexpressing cell lines. The results showed that cells with higher levels of NCAPH showed increased proliferation rates in the CCK‐8 and CFA assay. Furthermore, we found that the inhibition of NCAPH suppressed HCC proliferation in vivo and that the expression of the proliferation marker gene Ki‐67 was significantly decreased in NCAPH knockdown tumors.

Many studies have revealed that metastasis and recurrence are the most important reasons for the death of patients with HCC.^18^, ^19^ Therefore, we examined the function of NCAPH in migration and invasion. The results demonstrated that NCAPH could promote the ability of HCC cells to migrate and invade in vitro. EMT is the process by which epithelial cells transform into mesenchymal cells and plays an important role in HCC cell progression and metastasis.^20^, ^21^ Previous studies have revealed that various cytokines and growth factors could regulate the EMT pathway.^22^ In addition, slug, a member of snail, could repress the transcription of E‐cadherin.^23^ In our study, we found the NCAPH was associated with the adherens junction pathway. And the expression of NCAPH was positive correlated with slug in TCGA datasets. Furthermore, we detected the E‐cadherin, N‐cadherin, and vimentin expression in HCC tissues. We found that NCAPH expression were negative correlated with E‐cadherin, but positive correlated with N‐cadherin and vimentin. Moreover, we found that decreased NCAPH levels reduced the expression levels of mesenchymal markers while increasing the expression of epithelial markers in HCC cells. Furthermore, mesenchymal markers were increased but were epithelial markers decreased in the NCAPH‐overexpressing cell line. These findings suggest that NCAPH enhances migration and invasion through the induction of EMT in HCC.

In conclusion, our results provide evidence that NCAPH is overexpressed in HCC tissues and associated with poor prognosis. Moreover, NCAPH expression promotes HCC cell proliferation, migration and invasion through EMT. Therefore, NCAPH could be considered a novel prognostic biomarker and therapeutic target for HCC patients.

## CONFLICT OF INTERESTS

The authors declare that there are no conflict of interests.

## AUTHOR CONTRIBUTIONS

WJ, XH, and QZ designed the research. CS, SH, HW, RX, and LZ performed the research. SH and CS analyzed the data. CS wrote the manuscript. All authors read and approved the final manuscript.

## Supporting information


**Supplemental figure 1 The correlation of NCAPH with EMT markers.** A, NCAPH was correlated with gene sets in cell cycle, DNA replication and adherens junction in GSEA. B, The expression of NCAPH was positive correlated with slug in TCGA datasets (*R*=0.32, *p*=3.6e−10). C, NCAPH expression were negative correlated with E‐cadherin (*R* ^*2*^=0.2312, *p*=.0319). D,E, NCPAH expression were positive correlated with N‐cadherin (*R* ^*2*^=0.2385, *p*=.0289) and vimentin (*R* ^*2*^=0.2772, *p*=.0171)
