# Supplementary figures and images for: Non‐SMC condensin I complex subunit H enhances proliferation, migration, and invasion of hepatocellular carcinoma
Source: Mol Carcinog. 2019 Sep 15;58(12):2266–75. doi: 10.1002/mc.23114 (PMC6899668; doi:10.1002/mc.23114)

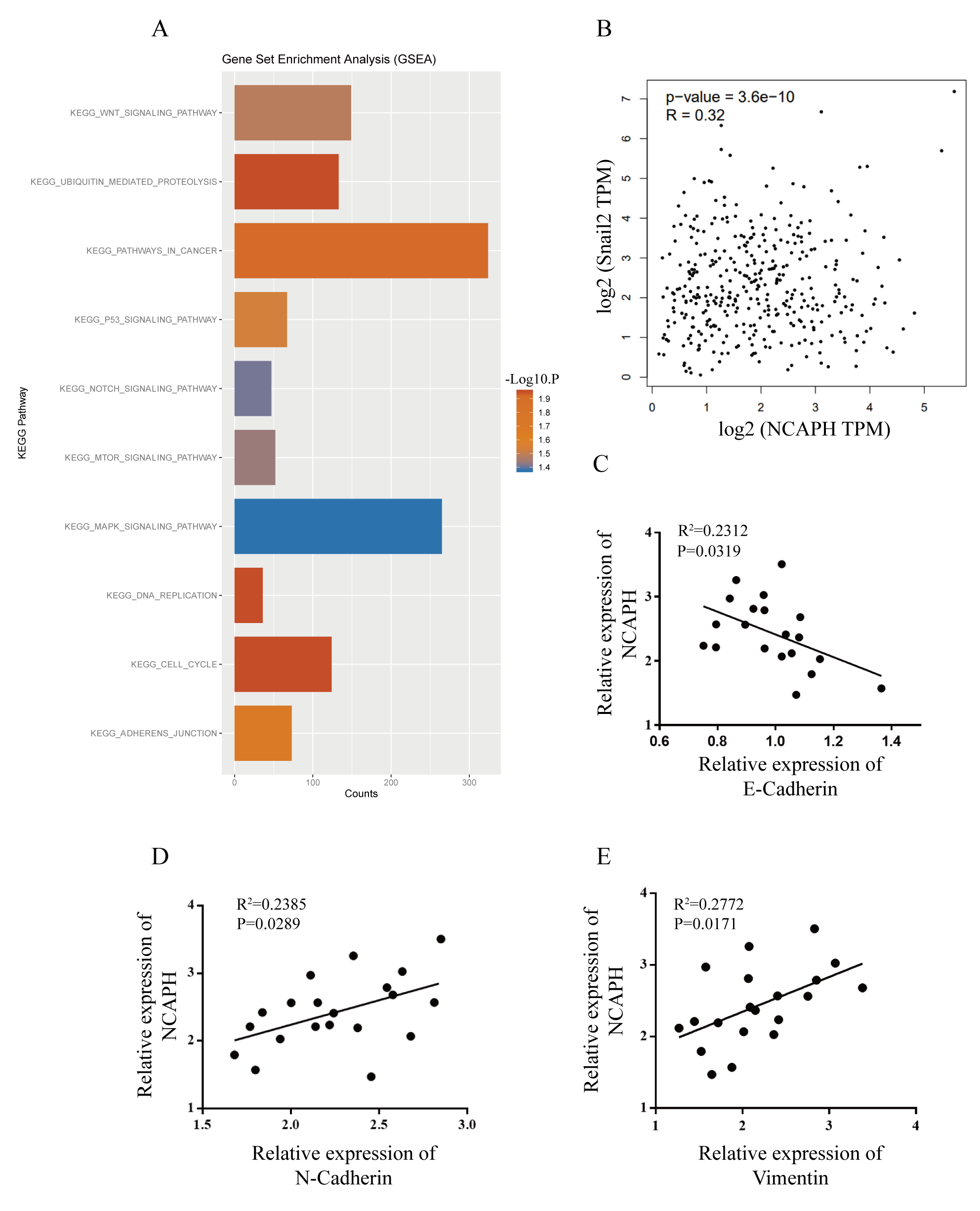

Supplement: Supplementary file 1 — Supplemental figure 1 The correlation of NCAPH with EMT markers. A, NCAPH was correlated with gene sets in cell cycle, DNA replication and adherens junction in GSEA. B, The expression of NCAPH was positive correlated with slug in TCGA datasets (R=0.32, p=3.6e−10). C, NCAPH expression were negative correlated with E‐cadherin (R 2=0.2312, p=.0319). D,E, NCPAH expression were positive correlated with N‐cadherin (R 2=0.2385, p=.0289) and vimentin (R 2=0.2772, p=.0171) [file MC-58-2266-s001.tif]
